# Night shifts, sleep deprivation, and attention performance in medical students

**DOI:** 10.5116/ijme.531a.f2c9

**Published:** 2014-03-29

**Authors:** Isabel Perez-Olmos, Milciades Ibanez-Pinilla

**Affiliations:** Research Center of the School of Medicine and Health Sciences, Rosario University, Colombia

**Keywords:** Sleep deprivation, nightshift work, attention performance, undergraduate medical students, Colombia

## Abstract

**Objectives:**

To determine attention performance of medical students after sleep deprivation due to night shift work.

**Methods:**

Prospective cohort design. All seventh, eighth and ninth semester students were invited to participate (n= 209). The effectiveness and concentration indices (d2 Test for attention, dependent variable) from 180 students at 3 evaluations during the semester were compared. Eighth and ninth semester students underwent their second evaluation after a night shift. The independent variables were nocturnal sleep measurements.

**Results:**

No differences in nocturnal sleep hours during the previous week (p=0.966), sleep deprivation (p=0.703) or effectiveness in the d2 Test (p=0.428) were found between the groups at the beginning of the semester. At the beginning and the end of the semester, the d2 Test results were not different between groups (p=0.410, p=0.394) respectively. The second evaluation showed greater sleep deprivation in students with night shift work (p<0.001). The sleep deprived students had lower concentration indices (p<0.001).The differences were associated with the magnitude of sleep deprivation (p=0.008). Multivariate regression analysis showed that attention performance was explained by sleep deprivation due to night shift work, adjusting for age and gender. Students with sleep deprivation had worse concentration than those without.

**Conclusions:**

Sleep deprivation due to night shift work in medical students had a negative impact on their attention performance. Medical educators should address these potential negative learning and patient care consequences of sleep deprivation in medical students due to night shifts.

## Introduction

Many authors have emphasized the importance of sleep for optimal cognitive and emotional performance, learning, memory consolidation, affective regulation,^1^^-^^4^ attention, and concentration,^5^among other related aspects.^6^^-^^8^ Moreover, some studies indicate that hippocampal neurogenesis, especially in the dentate gyrus, involved in learning and memory processes, is considered to be sleep dependent.^9^^-^^11^ Despite these evidences, some other authors have questioned the role of sleep in memory consolidation.^12^Scientific literature has shown that occupational activities with shifts that alter circadian sleep/wakefulness cycles lead to sleep deprivation (SD). In humans, sleep deprivation is associated with diurnal somnolence;^13^ attention, memory, and learning process deficits; and, in general, alterations in cognitive performance.^14^^,^^15^ It has also been shown that when SD is cumulative, there can be negative consequences for general health and productivity, as well as an increased risk of accidents.^16^ Some authors point out the need to take into account individual differences in the effects of SD, since there are indications of systematic features of individual vulnerability which warrant better definition and more research.^17^Research animal models have been created to facilitate the understanding of the neurobiological mechanisms underlying sleep/wakefulness cycles, altered sleep patterns, and cognitive deficits resulting from SD and fragmented sleep, which cannot be explored in human subjects.^15^ Notwithstanding these considerations, SD is inherent to medical education and clinical practice, even though health care is the main objective of the profession. Paradoxically, the negative consequences of SD in medical students have not been considered in some countries. It is thus important to determine the implications of sleep deprivation resulting from night-time shifts on daytime cognitive performance, learning, patient care, and in general the quality of life of health professionals, particularly medical students during their professional education. This is especially so, given the high academic demands and optimal patient care expected from medical students and medical professionals.^14^^,^^18^^-^^24^ Potential errors are an inherent risk of medical activities; therefore, it is essential not to overlook the possibility of prevention if they might be related to SD, associated with obligatory night shifts.^19^^-^^23^^,^^25^^,^^26^Almost a decade ago, the US Accreditation Council of Graduate Medical Education (ACGME) established norms which set 80 as the maximum number of weekly work hours, with no more than 24 hours of continuous work activity, and shifts no more often than every three days. Although these measures have improved the conditions according to some studies,^27^ others suggest that they are insufficient in completely preventing the negative effects of SD in medical residents or practicing physicians,^19^^,^^21^^,^^23^^,^^25^ as Philibert also showed in a meta-analysis.^22^In many countries, medical students do not have night shifts that might involve having SD with potential negative consequences for their learning process. Nevertheless, in Colombia and other developing countries, night shifts are frequent in undergraduate medical student clinical practices, but the suspected negative impact of the SD in those medical students has not been evaluated. This research project addresses the perceived need of assessing the negative impact on attention following sleep deprivation of medical students enrolled in their last clinical semesters at our university.The main objective of this study was to evaluate the negative effects on daytime attention performance resulting from SD due to night shift work within the clinical practice of medical students.

## Methods

### Study design

We performed a longitudinal, prospective cohort study with 3 measuring points during an academic semester, including 3 different groups (seventh, eighth and ninth semesters; roughly 4th to 5th year medical students in other countries) of medical students at the Rosario University. Eighth and ninth semester students underwent night shifts as part of their clinical rotations (exposed group), and seventh semester students were a control group, without night shifts during the semester (non-exposed group).

### Participants

The medical program consists of 12 academic semesters (6 years). The students begin clinical night shifts in semesters VIII and IX (4th to 5th year medical students). The seventh semester students participated as a control group. The population comprised a total of 209 students. Criteria for inclusion were: to be a seventh, eighth or ninth semester medical student, over the age of 18, who voluntarily agreed to participate in the study. Students with incomplete questionnaires or attention performance d2 Tests, with serious attention deficits in the first measurement, or who abandoned the program or the semester for whatever reason, were excluded.

### Sample size and sampling method

Selected target population included all the registered students in semesters VII to IX (n=209). Response rate was 86%, so the study sample size was 180 students. The group with night shifts during the semester consisted of 110 students (58 in eighth semester, and 52 in ninth semester); the control group comprised 70 seventh semester students.

### Procedure

Two assessment instruments were applied to all the participants at 3 points during the academic semester: the validated d2 Test for attention and concentration performance, and a sleep questionnaire. The d2 Test is a validated neurocognitive assessment of selective attention and concentration, manifested by the capacity to attend selectively to some relevant aspects of a task while simultaneously ignoring aspects defined as not relevant; moreover, the test must be done rapidly and precisely. It consists of a template with 14 lines, each of which contains 47 characters (for a total of 658 stimuli in the test). The characters have the form of a “d” or “p” marked with one, two, three, or four, quotation marks, above, below, or both above and below the letter. The task involves reviewing the lines during a limited time of 20 seconds per line, marking with a cross the “d’s” that have two marks (relevant stimulus), while ignoring the other, different characters (irrelevant stimuli). The d2 Test has been demonstrated to be highly reliable and valid.^28^^,^^29^ The application, either to an individual or to a group, using self-correcting templates, takes between 8 and 10 minutes. It is designed for adults and young people 8 years old and above. Due to its characteristics, it has been widely used in basic and applied research. It was developed and validated initially during the early seventies in German-speaking European countries, and has since become one of the most important tests for evaluating attention.^28^^,^^29^The study questionnaire was a self-administered survey. It compiled information about the independent variables, academic semester, sex and age of the medical students, normal sleep pattern characteristics such as number of hours of nocturnal sleep required for the student to wake up fully refreshed physically and mentally, daytime naps, the number of hours of nocturnal sleep during the previous week, sleep-wake cycle influencing factors, current stressors, name of the clinical rotation, consumption of psychoactive substances, neurological and psychiatric disorders, and drugs used. The questionnaire also included Epworth’s Sleepiness Scale, which has been validated in Colombia.^30^^,^^31^

### Data collection

At the beginning of the academic semester, the investigators invited all the students in semesters VII, VIII, and IX to participate in the project, after presenting them with information about the objectives, methods, and details of the study.All of the measurements (sleep questionnaire, and d2 Test) were taken early in the morning between 7 and 10 AM at the latest. The study was carried out at three points during the semester: 1) In order to guarantee baseline population homogeneity on dependent and independent variables, the study started with a baseline measurement taken collectively at the beginning of the semester. 2) In the middle of the academic semester each participant who was sleep-deprived as a result of night shifts was evaluated individually (semesters VIII and IX); while the control group (semester VII) was evaluated simultaneously. 3) The final evaluation was done at the end of the semester without regard to sleep deprivation from any cause.In the second evaluation, eighth and ninth semester students were evaluated under conditions of sleep deprivation after a night shift; this condition might result in a participant bias for precisely this reason. We attempted to control for this bias by motivating the participants, stimulating them to complete the d2 Test application in the same way that they had done at the beginning of the semester and not sloppily because they were tired or sleepy. We were able to do this by giving the test, which only takes 5 to 10 minutes, early in the morning and moreover letting them know that after the test they could go home and rest instead of continuing with clinical activities. They were motivated to contribute high quality data that would guarantee the validity of the study results.

### Statistical analysis

The data from the d2 Test and the sleep questionnaire were entered in a database in Excel (Microsoft Office); the statistical analysis was carried out using the SPSS Program, version 20.0, for Windows, and Stata Program. The statistical tests were evaluated with a 5% level of significance (p<0.05).

### Variables

The dependent variable in this study was the performance on the d2 attention test, specifically two quantitative measures: 1) concentration index (total number of correctly processed stimuli minus total number of incorrectly processed stimuli) and 2) total efficiency index (total of answers minus errors (commissions and omissions)).^28^^,^^29^ These measures were obtained for each of the 3 assessments performed during the academic semester.The independent variables were gender, age, academic semester, sleep profile measures from the three semester point evaluations, and the sleep deprivation hours due to night shifts registered in the second evaluation for eighth and ninth semester students.The students’ nocturnal sleep profiles were characterized by two summary independent variables for each of the study’s three phases: the mean number of hours of nocturnal sleep during the week prior to the assessment, and the mean number of SD hours per night according to the self-perceived need of each student.In order to guarantee the validity of statistical analysis and comparisons among groups, as has been highlighted elsewhere,^32^ the normality of quantitative variable distributions was evaluated with the Kolmogorov-Smirnov and Shapiro-Wilk tests. If a non-normal distribution was found, non-parametric tests were applied.The mean number of hours of nocturnal sleep during the previous week was evaluated for the three periods with the ANOVA of repeated parametric measures, and the multiple variables comparison with the Bonferroni Test.Mean nocturnal sleep deficit and the mean number of hours of sleep in the previous week at each measurement point was compared between the group with night shifts and the control group, using Student’s t-test for independent groups, with or without homogeneous variance (previously assessed with the Levene test); in cases of non-normal distribution, the nonparametric Mann-Whitney *U* test was used.In order to complete the nocturnal sleep deprivation determination, we constructed two independent variables taking into account whether this was due to night shift work: the sleep deficit as perceived by the participating student, and the mean number of hours of nocturnal sleep in the previous week. With the Kruskal-Wallis nonparametric ANOVA test, we compared the median performances on the d2 Test, specifically the index of concentration, and the total efficiency (total of correct answers), among the sleep deprived groups, and according to the mean hours of nocturnal sleep in the previous week.Categories of “sleep deprivation associated with night shift work” were defined based on the difference between the mean number of hours of nocturnal sleep in the previous week and the number of hours required each night, as perceived by the student. This independent variable was calculated for each of the study´s three evaluations and was classified as follows: 1) No nocturnal sleep deficit, 2) A mean of 0.1-2 hours of nocturnal sleep deficit, and 3) >2 hours of nocturnal sleep deficit. These cut-off categories resulted from the bivariate analysis and frequency distribution of the variable of interest.Finally, we performed a multivariate regression analysis using the nonparametric model of the median to determine the dependent variable (d2 Test performance) in conditions of nocturnal sleep deprivation due to night shift work, adjusting for the variables of age, gender, and mean hours of nocturnal sleep in the previous week. Collinearity among independent variables was evaluated to select the best multivariate regression model for determining the relationships among the variables of interest.study was approved by the Research Ethics Committee of the Rosario University Medical School. A verbal informed consent was obtained after a detailed presentation of the project to the medical students. The researchers were not teachers of the students during the academic semester when the study was carried out. The student’s right to decide voluntarily whether to participate or not was respected, and the information collected was treated as confidential. At the end of the study, each student received his/her personal results privately.

## Results

### General characteristics

The study sample was made up of 180 medical students from seventh through ninth semesters; 63.9% (115) of these were women. Their ages ranged from 18-26 years, with an average of 21.3±1.4 years, and a median of 21 years at the beginning of the study. Mean number of hours of nocturnal sleep during the previous week at the start of the semester (baseline) was significantly greater than in the second and third evaluations (p<0.001, p<0.001) respectively. See Table 1.

**Table 1:** Three semesters nocturnal sleep and sleep deprivation evaluations of undergraduate medical students, Universidad del Rosario, Bogotá, Colombia, 2009 (N=180)

Sleep deprivation groups	Nocturnal sleep hours during the previous week			Sleep deprivation hours per night		
	evaluation 1	evaluation 2	evaluation 3	evaluation 1	evaluation 2	evaluation 3
Night shift before evaluation 2	n=110	n=105	n=110	n=109	n=98	n=101
Mean	7.96	5.63	6.90	0.04	2.13	0.85
Median	8.14	5.36	6.86	0.00	1.71	0.71
SD	1.77	1.33	1.58	1.90	2.16	2.03
Without night shift	n=70	n=69	n=70	n=69	n=65	n=67
Mean	7.97	6.14	5.44	-0.07	0.93	1.88
Median	8.14	6.14	5.43	0.14	0.71	1.86
SD	1.52	1.54	0.96	1.87	1.63	1.53
Total	n=180	n=174	n=180	n=178	n=163	n=168
Mean	7.96	5.83	6.33	0.00	1.65	1.26
Median	8.14	5.75	6.14	0.00	1.57	1.14
SD	1.68	1.44	1.54	1.88	2.05	1.91

### Comparison between groups according to night shift exposure

The group with night shifts during the semester (exposed) consisted of 110 students (58 in the eighth and 52 in the ninth semester); the non-exposed group comprised 70 seventh semester students. There was no significant difference in gender distribution between groups (χ^2^=2.82, p=0.093). The exposed group was significantly older (21.6±1.45, med=21.5) than the unexposed group (20.9±1.19, med=21) (Mann-Whitney *U*=2628, p<0.001).At the beginning of the semester, there was no significant difference between groups in the mean number of nocturnal sleep hours during the previous week (*t*=0.043, p=0.966). In the second evaluation, this value was significantly lower in the group with sleep deprivation due to night shifts (p=0.001) in comparison with the control group. At the end of the semester, the group of seventh semester students had fewer hours of nocturnal sleep (p<0.001).There were similar results in the distribution of sleep deprivation calculated at the three evaluation points during the semester: at the beginning (*t*=0.382, p=0.703), in the middle (*t*=3.807, p<0.001), and at the end of semester (*t*=3.53, p<0.001).

### Relation between nocturnal sleep deprivation and performance on the d2 Test (Concentration index and total effectiveness index)

There was no difference in d2 Test concentration index between groups at the beginning or at the end of the semester (p=0.41, p=0.394) respectively. There was, however, a significant difference in the second evaluation: the sleep-deprived group showed a significantly lower median concentration index than the control group (70 vs. 90) (Mann-Whitney *U*=2538, p<0.001).The results for the total number of correct answers given (effectiveness in the d2 Test) were similar: no differences at the beginning and the end of the semester, and a highly significant difference in the second evaluation (Mann-Whitney *U*=2569, p<0.001); the sleep-deprived group showed a significantly lower median. A significant relationship to the magnitude of the sleep deficit, as measured by the three defined categories (χ^2^=15.61, p=0.008) was found; the most sleep-deprived group (>2 hours) scored lowest and the group without sleep deprivation scored highest in concentration (Table 2).

**Table 2:** Attention performance on d2 Test and nocturnal sleep and sleep deprivation, second evaluation of medical students, Universidad del Rosario, Colombia, 2009 (N=174)

Sleep deprivation among night-shifts groups (N=162)	Statistics	Total effectiveness d2 Test – 2^nd^ evaluation	Concentration index d2 Test - 2^nd^ evaluation
Group without night-shifts (n=65)			
hr(s) <= 0 (n=19)	Mean	89.68	89.47
	Median	95	95
	SD	10.46	9.88
hr(s) = 0.01- 2 (n=30)	Mean	81.33	79.27
	Median	90	90
	SD	20.35	21.43
hr(s) > 2 (n=16)	Mean	82.56	76.69
	Median	92.5	87.5
	SD	23.88	29.64
Night-shifts group (n=97)			
hr(s) <= 0 (n=11)	Mean	80.36	76.09
	Median	90	90
	SD	24.12	28.29
hr(s) = 0.01- 2 (n=41)	Mean	71.29	67.63
	Median	80	75
	SD	24.7	27.07
hr(s) = > 2 (n=45)	Mean	71.13	66.09
	Median	85	70
	SD	28.29	30.22

Nocturnal sleep during the previous week among night shift groups (N=174)	Statistics	Total effectiveness d2 Test –2^nd^ evaluation	Concentration index d2 Test –2^nd^ evaluation
Group without night-shifts (n=69)			
hr(s) => 6.5 (n= 32)	Mean	83.31	82.28
	Median	90	87.5
	SD	18.76	19.62
hr(s) = 5 - 6.4 (n=23)	Mean	86.57	82.83
	Median	95	95
	SD	17.71	23.38
hr(s) < = 5 (n=14)	Mean	83.86	80.5
	Median	92.5	87.5
	SD	21.16	22.16
Night-shift group (n=105)			
hr(s) => 6.5 (n=25)	Mean	69.24	66.36
	Median	80	65
	SD	28.81	28.5
hr(s) = 5 - 6.4 (n=39)	Mean	77.26	72.97
	Median	85	80
	SD	21.26	23.9
hr(s) < = 5 (n=41)	Mean	66.29	61.29
	Median	80	70
	SD	29.49	32.35

We found a similar relationship for the mean number of hours of sleep during the previous week, classified in three categories (<= 5 hours, 5.01-6.49 hours and >=6.5 hours). The sleep deprived group with the fewest hours of sleep in the previous week had lower concentration scores, while the group with the most sleep hours scored higher (χ^2^=15.03, p=0.010). The results for the effectiveness index on the d2 (total number of correct answers) were consistent: the sleep-deprived group scored lower (p=0.013) in proportion to the mean number of nocturnal sleep hours during the previous week (χ^2^=14.84, p=0.011) (Table 2).

### Multivariate regression analysis

The concentration index and the effectiveness on the d2 Test were explained by sleep deprivation due to medical night shift work, adjusted for the student’s age and gender. The sleep-deprived students showed poorer concentration than controls. Younger students from the control group without sleep deprivation due to night shift work scored significantly better on both concentration and total effectiveness (Table 3).

**Table 3:** Multivariate regression analysis for attention performance on d2 Test as dependent variable. Medical students, Universidad del Rosario, Colombia, 2009* (N=179)

Concentration index on the d2 Test (N=179)	β Coefficient	p	β CI 95%	
			Lower	Upper
Night shift	-11.5	0.001	-18.07	-4.92
Age	-4.5	0.000	-6.77	-2.22
Gender	-6.5	0.055	-13.13	0.13
Constant	184.5	0.000	136.79	232.2
Concentration index on the d2 Test (N=174)				
Night shift and mean hours of nocturnal sleep during the previous week	-3	0.004	-5.05	-0.94
Age	-5	0.001	-7.88	-2.11
Gender	-5	0.244	-13.44	3.44
Constant	198	0.000	137.08	258.91
Effectiveness index on the d2 Test (N=179)				
Night shift	-8	0.009	-14	-1.99
Age	-3	0.006	-5.11	-0.88
Gender	-5	0.104	-11.03	1.03
Constant	156	0.000	111.88	200.11
Effectiveness index on the d2 Test (N=179)				
Night shift and mean hours of nocturnal sleep during the previous week	-1.58	0.007	-2.71	-0.44
Age	-3.66	0.000	-5.22	-2.1
Gender	-4.75	0.041	-9.3	-0.19
Constant	171.5	0.000	138.62	204.37

^*Nonparametric model of the median.^

The concentration and the effectiveness index on the d2 Test, controlled for age, sex, and the number of sleep hours during the previous week, were significantly associated with the exposure to sleep deprivation due to night shift work. Those who combined night shift work with lower average number of sleep hours during the previous week scored significantly poorer for both concentration and effectiveness on the d2 Test. Age showed an inverse behaviour (Table 3).

## Discussion

Female students, outnumbered men in the three groups evaluated (63.9%); this tendency has been shown previously for undergraduate medical students in Colombia.^33^The average age at the beginning of the study was 21.3 ± 1.4 years, and the median and mode were 21 years. As expected, the first evaluation at the beginning of the semester did not show differences between groups on d2 attention test performance or in sleep profiles, thus guaranteeing the homogeneity and comparability of the study groups.The scientific literature contains much evidence of the negative effects of sleep deprivation on cognitive performance, such as slowed thought, alterations in attention and memory, deficits in psychomotor tasks, concentration difficulties, and language and mood alteration. Nevertheless, these studies have been carried out on practicing physicians, postgraduate medical residents, and adults in nonmedical occupations who differed in age from this study’s sample population.^13^^,^^16^^,^^20^^-^^23^Similar studies on younger medical students are scarce; and in Hispanic and Latin American countries they are practically non-existent in the published literature.^34^^,^^35^

Studies such as the present one are important given the possibility that alterations in attention and concentration resulting from nocturnal sleep deprivation might adversely affect undergraduate medical students’ learning and academic performance, as well as the quality of patient care they provide in their clinical practices.^14^^,^^19^^,^^24^^,^^34^^,^^35^Studies on the effects of nocturnal sleep deprivation associated with medical students’ night shifts are rare, probably due to the fact that medical undergraduate student night shifts do not occur in most countries. In spite of the age differences in this study in comparison with the other studies cited, the findings on attention and concentration are consistent with the literature.^5^^,^^22^^,^^36^ Our findings confirm an immediate and quantifiable negative impact on undergraduate medical students’ attention and concentration the day after sleep deprivation associated with a medical night shift.Our results also confirm that the negative impact from SD is cumulative, as has been shown by other authors.^16^ This could explain why the most sleep-deprived group (>2 hours) scored lowest, and the group without sleep deprivation scored highest in concentration. Overall test performance, specifically in concentration and total effectiveness, showed a dose-response to the magnitude of the recent sleep deprivation as well as to the mean nocturnal sleep hours during the previous week.The consequences of sleep deprivation evaluated in residents of different medical specialties have led to the enactment of norms in the United States regulating the maximum number of work hours per week and the number of hours of continuous work in order to avoid acute and accumulated SD. Even so, it appears that these are insufficient in preventing negative effects on daytime performance.^19^^,^^37^^,^^38^This study provides new evidence of the short term impact of sleep deprivation on a population of younger undergraduate medical students whose age is different from that of populations evaluated by other authors.^13^^,^^14^^,^^16^^,^^24^^,^^36^ In accordance with the conclusions of Philibert’s meta-analysis,^22^ the implications of these results are the need for established limits to work hours, based on scientific evidence, as well as for increased efforts that will improve both patient safety and optimal student learning.Just 10 years ago, the need emerged to create an alliance among various organizations in the United States to establish a work agenda dealing with the problems in daytime performance resulting from sleep deprivation. It sought to avoid negative consequences of sleep deprivation on learning and patient care, and preventable errors in postgraduate medical education, but also to lead to an improved quality of life for medical residents. There are many studies still to be done to guarantee the fulfilment of these agreed-upon objectives.^22^^,^^25^Regarding the limitations of this study, one is in reference to the generalization of the results, due to the fact that all participating students are from a single medical school. Further studies with similar young medical student populations are needed in order to replicate these results. Although financial limitations prevented our use of objective sleep measures such as actigraphy, the data obtained showed a short term negative impact on attention performance from sleep deprivation due to night shift work. We do not think that the results could be better explained by participants’ subjective biases.A limitation in many follow-up studies is participant losses; in this study, the response rate was 86%. The 14% that did not participate is a relatively low proportion, given the habitual difficulties of this type of study. Additionally, in this study we managed to complete a follow-up of 96.7% of the participants (174 out of an initial sample of 180) this loss of 3.3% during the study is exceptionally low, especially in the context of the limited time availability of university students carrying a heavy academic load. We have no reason to suspect a sample bias that might have influenced the results of the study.Despite the mentioned limitations of this study, the data obtained allowed the detection of statistically significant differences in the attention performance of groups exposed to SD due to medical night shifts, the main dependent variable evaluated in this study.

## Conclusions

Sleep deprivation resulting from night shifts in clinical rotations had a negative impact on medical student selective attention and concentration performance. This negative effect showed a dose response: greater deprivation was associated with worse concentration and selective attention.This may have negative consequences for medical learning and patient care. Medical educators should know that sleep deprivation due to night shifts alters the daytime concentration of their students.This evidence should be taken into account in the academic programming of clinical activities in order to minimize the negative consequences of sleep deprivation that result from medical students who are required to work night shifts.The results of this study add evidence of the negative impact sleep deprivation has on attention performance of a young population of medical students. This deprivation has potential negative consequences on learning, supporting the need for norms that regulate night shift work in medical student programs.

## 

### Conflict of Interest

The authors declare that they have no conflict of interest.
